# Long-Term Evolution of Microstructure, Density, and Yield Strength of Pure Lead After Solidification Under Different Cooling Rates

**DOI:** 10.3390/ma19122530

**Published:** 2026-06-11

**Authors:** Bingjie Wu, Hailuo Zhong, Weibing Liao, Mingdong Zhu, Yuanyuan Dong, Xi Huang

**Affiliations:** 1 Science and Technology on Reactor System Design Technology Laboratory, Chengdu 610041, China; wubingjie204@npic.ac.cn (B.W.); zhumingdong@npic.ac.cn (M.Z.); drsi-d20001@npic.ac.cn (Y.D.); 2 College of Physics and Optoelectronic Engineering, Shenzhen University, Shenzhen 518060, China; 2017183017@email.szu.edu.cn (H.Z.); liaowb@szu.edu.cn (W.L.); 3 Shenzhen Key Laboratory of Nuclear and Radiation Safety, Shenzhen 518060, China

**Keywords:** pure lead, long-term evolution, cooling rate, mechanical properties, microstructure evolution, lead-cooled fast reactor

## Abstract

Lead-based alloy has received widespread attention as a coolant in nuclear reactors. However, there is limited research on pure lead after solidification. In this study, a systematic investigation was conducted on the long-term evolution of the microstructure and physical properties of pure lead samples solidified under different cooling rates, with a comparative analysis against of lead–bismuth eutectic (LBE). Microscopic detection (using optical and electron microscopes), density measurement, and compressive mechanical testing were carried out. The study results show that during the long-term evolution process after solidification (at room temperature of 27 °C), pure lead samples spontaneously undergo recovery and recrystallization, with larger grain size and more uniform microstructure. The density of samples remains within a stable range. The yield strength of samples after solidification will gradually decrease over time. For example, after 180 days of evolution, the yield strength of the rapidly cooled sample (10 K/min) decreased from 4.879 MPa to 3.766 MPa.

## 1. Introduction

### 1.1. Lead and LBE as Reactor Coolants

Traditional nuclear reactors have decades of operational experience, and rich experience has been accumulated in every aspect from design and construction to operation. The fourth-generation nuclear energy system, which covers reactors and their fuel cycles, can be briefly referred to as fourth-generation reactors [1]. It represents a major trend and technological frontier in the field of nuclear energy.

The system includes six candidate reactor types: the Very High Temperature Gas-Cooled Reactor (VHTR), Sodium-Cooled Fast Reactor (SFR), Molten Salt Reactor (MSR), Lead-Cooled Fast Reactor (LFR), Gas-Cooled Fast Reactor (GFR), and the Supercritical Water-Cooled Reactor (SCWR) [2,3,4,5,6,7,8,9]. Among these, the Lead-Cooled Fast Reactor (LFR), with its unique technical advantages and development potential, is widely regarded as one of the most promising reactor types. It is expected to achieve industrial demonstration first and become a pioneer of the fourth-generation nuclear energy systems [10]. Lead-based alloys, such as lead–bismuth eutectic (LBE) and pure lead, are used as coolants in fourth-generation lead-cooled fast reactors [11,12]. By optimizing the structural design and improving operational efficiency, the costs of construction and operation can be effectively reduced, making nuclear power generation a more competitive choice for clean energy [13,14]. LBE has a low melting point, which allows the nuclear reactor system to operate at lower temperatures and pressures, thereby reducing the safety hazards to the reactor components caused by high-temperature and high-pressure operations. Pure lead possesses excellent thermal conductivity, enabling it to efficiently conduct heat generated by the core, thereby improving the reactor’s thermal transfer efficiency. Moreover, its high boiling point (approximately 1620 °C) prevents the risk of coolant boiling. The high density of pure lead (approximately 11.34 g/cm^3^) plays a key role in ensuring nuclear safety. Firstly, the high-density characteristic endows lead-cooled fast reactors with the ability to prevent re-criticality under extreme accident conditions, thereby enhancing reactor safety. Secondly, the high density of pure lead renders it an effective shielding medium for nuclear radiation, such as gamma and X-rays, thereby reducing radiation leakage. Moreover, as an inert metal, pure lead reacts very little with water or air, avoiding the risks of vigorous chemical reactions and hydrogen generation. This characteristic not only enhances the operational safety of the reactor but also simplifies maintenance requirements. Additionally, the chemical stability of lead reduces the corrosion of the core structural materials, thereby extending the service life of the equipment.

### 1.2. Known Post-Solidification Evolution Behavior of LBE

**Figure 1:**
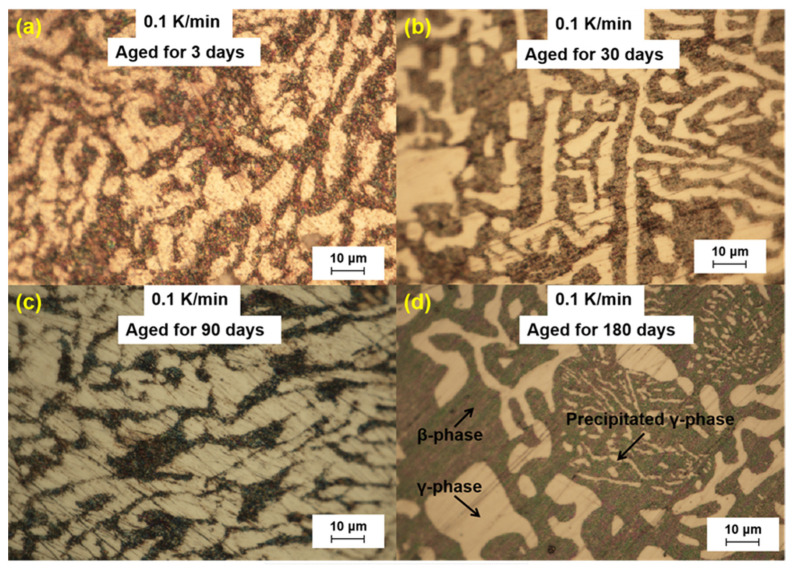
Optical microscope images of the microstructure evolution of LBE samples at different time points under a cooling rate of 0.1 K/min. (**a**) 0.1 K/min LBE samples were aged 3 d, (**b**) 0.1 K/min LBE samples were aged 30 d, (**c**) 0.1 K/min LBE samples were aged 90 d, (**d**) 0.1 K/min LBE samples were aged 180 d [31].

Liquid heavy-metal coolants offer numerous technical advantages. However, they also present a series of complex challenges during the actual service of reactors. Although LBE has many benefits as a coolant, it also has some drawbacks. For example, issues such as the corrosion of liquid LBE and volume expansion during the evolution process after solidification can have a certain degree of impact on the structural materials of the reactor [15,16,17,18,19,20,21,22,23,24,25,26,27,28,29,30,31,32,33,34,35]. Liquid LBE corrosion refers to the phenomenon of material performance degradation caused by element precipitation and reaction between LBE and steel after contact [15]. The corrosion mechanisms include oxidation, dissolution, erosion, impingement, and abrasion [15,16,17,18,19,20]. Steel materials such as Fe, Cr, and Ni can dissolve in LBE, leading to severe corrosion of the structural materials of the reactor and mechanical pump blades. Furthermore, LBE produces polonium-210 after neutron irradiation, a highly toxic substance with volatility, radioactivity, and a long half-life, which significantly increases the operational and maintenance difficulties of the reactor. The literature indicates that in the early stage of solid phase formation, the volume of LBE will shrink, and then gradually expand during the evolution process [17,18,19,20]. Figure 1 shows the microstructural evolution of the LBE samples at different evolution times at a cooling rate of 0.1 K/min. After 180 days of evolution, a phase transformation from the β-phase to the γ-phase occurs within the LBE [31]. Conversely, the absence of Bi in pure lead implies that no radioactive and highly toxic Po-210 is produced under neutron irradiation, which reduces the operational and maintenance challenges of the reactor. Furthermore, pure lead exerts a relatively lower corrosion effect on the structural materials of the reactor, which helps to extend the service life of the reactor. Therefore, pure lead can also serve as an excellent lead-based coolant.

### 1.3. Lack of Comparable Long-Term Studies on Pure Lead

The solidification/melting of liquid heavy metals and their subsequent evolution processes are significantly influenced by experimental conditions. Variations in the heating/cooling rates or changes in the environmental temperature during the evolution process can lead to alterations in the physical properties of the heavy metal, such as its density and yield strength, which will exhibit different patterns of change under different experimental conditions. Although LBE has been widely studied, the long-term evolution of solidified pure lead under different cooling conditions remains insufficiently understood. Therefore, further investigations are warranted. This study aims to systematically investigate the long-term evolution of the microstructure and yield strength of pure lead after solidification by controlling its solidification cooling conditions and evolution time.

To reveal the commonalities, differences, and potential mechanisms of the long-term evolution process of pure lead and lead bismuth eutectic alloys (LBE) after solidification, systematic solidification experiments were conducted on pure lead at different cooling rates. After the sample is cooled, it is stored at room temperature (27 °C) to systematically characterize the microstructure, density, and mechanical properties of pure lead under different experimental conditions, with a focus on exploring the evolution laws of microstructure such as grain size distribution and morphology characteristics during its long-term evolution process; Simultaneously quantitatively test the evolution law of physical parameters such as yield strength and density over time. Based on multiple experimental characterization results, elucidate the regulatory mechanism of cooling rate and evolution time on the microstructure and mechanical properties of pure lead. Finally, under the premise of strictly unifying sample preparation, processing technology, and storage environment conditions at room temperature (27 °C), the experimental results of pure lead were compared and analyzed with existing LBE data, aiming to clarify the common characteristics and essential differences in two typical lead-based coolants in the long-term evolution process. Relevant research can provide basic data support for the evaluation of coolant organization stability and structural safety of lead-based reactors under shutdown and accident conditions.

## 2. Materials and Methods

### 2.1. Sample Preparation

In order to obtain pure lead samples suitable for the experiment, a sample preparation device was first set up. The container size of the sample preparation device is: inner diameter 90 mm, height 200 mm, wall thickness 5 mm. It is filled with cooling oil, with a height of 180 mm inside. To achieve precise control of the cooling rate, a comparison sample tube was set up, and a thermocouple was inserted into the comparison tube for temperature control. The sample preparation device is connected to the collector, and the experimental data is collected using computer software. The entire sample preparation process was carried out under a sealed condition.

The pure lead samples were prepared using four different cooling rates: 10, 5, 1, and 0.1 K/min. The starting temperature for sample preparation was 623 K (above the melting point of 600.63 K), and the final temperature was 300 K. Among them, fast cooling rates (5 K/min and 10 K/min) and slow cooling rates (0.1 K/min and 1 K/min) were set. These four rates cover the cooling range from extremely slow cooling to faster cooling, in order to systematically explore the effects of cooling rates on the solidification behavior, grain size, and grain boundary morphology of pure lead; adapt to practical engineering application scenarios; and achieve reasonable simulation of temperature decay and the cooling process of core lead-based coolant under normal shutdown or accident conditions. In terms of time scale selection, this study differentiated the time points for compressive mechanical performance testing and microscopic observation based on the core requirements of different testing purposes. The specific basis is as follows: (a) For compressive mechanical performance testing, the core focus of this study is the dynamic change law of pure lead mechanical performance in the long-term evolution process. Therefore, four characteristic time points of 3, 15, 90, and 180 days are selected for targeted testing. Through phased testing, the system captures the evolution trend of mechanical performance over time, ensuring that the performance change characteristics in the evolution process can be accurately reflected. (b) The core objective of microscopic observation is to determine the range of grain size changes in pure lead during long-term evolution, without the need to track its dynamic evolution process. After solidification, pure lead is stored at room temperature, and under these conditions, the material will spontaneously undergo recovery and recrystallization behavior inside. Due to the short duration of this process, the main purpose of microscopic characterization is to explore the regulatory effects of cooling rate and evolution time on the grain size of pure lead. Therefore, the selection of time scale is relatively loose, and randomly selecting three time points of 3, 15, and 360 days for testing can meet the research needs.

### 2.2. Mechanical Testing

**Figure 2:**
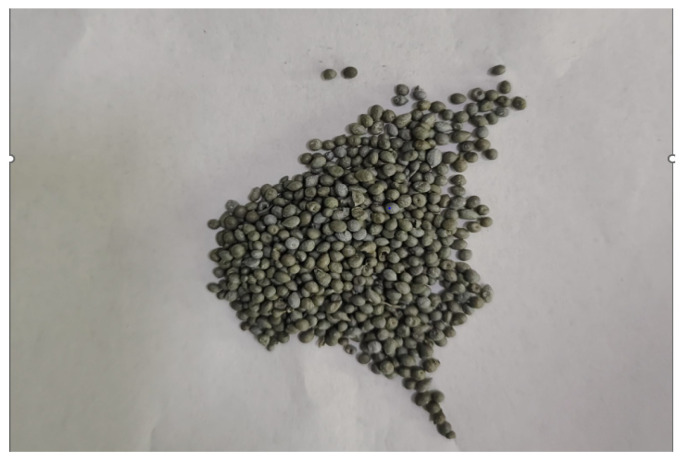
Raw materials for the preparation of pure lead samples.

Four cylindrical pure lead samples with a diameter of 15 mm and length of 12 cm were prepared using the experimental apparatus. After preparation, store the samples in a vacuum box in the laboratory at room temperature (27 °C). The raw material used to prepare the pure lead samples consisted of 99.9963 wt% Pb, as shown in Figure 2. Subsequently, compressive mechanical property tests were conducted at four different time points. The selected time points were 3, 15, 90, and 180 days after the completion of sample preparation. The samples prepared at different cooling rates were divided into four groups: 0.1 K/min for group 1 (G1), 1 K/min for group 2 (G2), 5 K/min for group 3 (G3), and 10 K/min for group 4 (G4). At a compression rate of 10^−3^ s^−1^, the compressive mechanical properties of pure lead samples were tested using a compression-tension testing machine (SHIMADZU AGX-V 100 KN, Kyoto, Japan). This experiment consists of 4 rounds of timing testing, including 4 temperature conditions, with 3 repeated tests conducted for each temperature. The samples used for testing are cylindrical samples with a diameter of 5 mm and a height of 5 mm.

### 2.3. Microscopic Observation

The time points for microstructure observation were 3 days, 15 days and 360 days after the sample solidification. An optical microscope (Nikon LV100ND/LV100NDA industrial metallurgical microscope, Tokyo, Japan) and a scanning electron microscope (SEM) were used to observe the morphological characteristics of the pure lead samples at different evolution stages. The metallographic preparation process mainly consists of two steps: polishing and chemical etching. First, the observation surfaces of all samples, which are 10 mm × 10 mm in size, are mechanically ground and polished. Then, chemical etching is carried out using the wiping method, with the etching agent being a mixed solution of freshly prepared glacial acetic acid and 30% hydrogen peroxide. After the etching agent is evenly wiped on the sample surface for 90 s, it is immediately rinsed with ethanol and dried with a fan. The treated surface becomes the target area for subsequent optical microscope and SEM observation.

### 2.4. Density Measurement

To investigate the variation in density over time in solidified pure lead samples, density measurements were conducted using the conventional Archimedes method. The period for density measurement ranges from any time point within the range of 50 to 2700 h of the long-term evolution process. Prior to measurement, the surfaces of the pure lead samples were polished, and the surface oxide films were removed by cleaning with ethanol and ultrasonication.

## 3. Results and Discussion

### 3.1. Stability of Density

**Figure 3:**
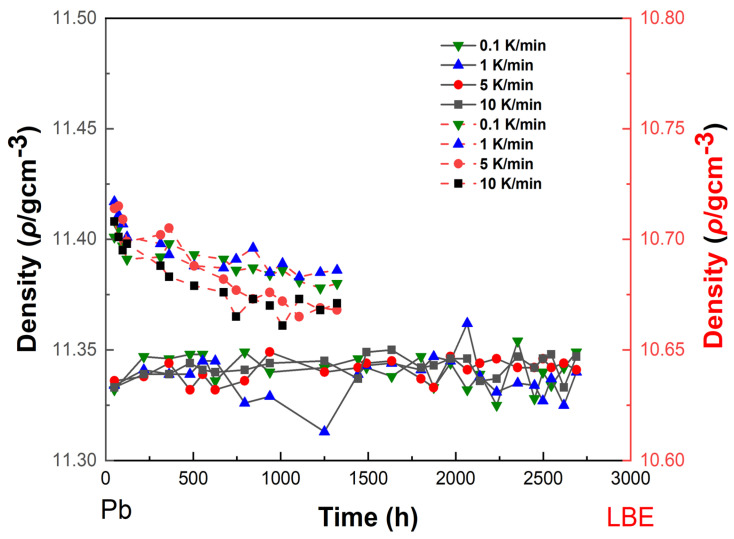
Density results of pure lead and LBE samples at different cooling rates.

Figure 3 shows the variation in density over time for the pure lead and LBE samples obtained at different cooling rates. The black curve represents the density of pure lead, and the red dashed line represents the density of LBE. The zerotime point corresponds to the completion of the sample cooling. The densities of the pure lead samples at different cooling conditions showed a similar trend. The density measured for the first time was approximately 11.33 g/cm^3^, and the final measurement was approximately 11.34 g/cm^3^, at which point the densities of all samples at different cooling conditions stabilized. Throughout the entire measurement process, the maximum fluctuation range of the density values was within 0.012 ± 0.003 g/cm^3^.

The density of the pure lead samples exhibited a relatively stable state under the relevant experimental conditions. The changes in cooling rate and long-term evolution have little impact on it. The density of pure lead demonstrates good stability at normal temperatures and pressures. On the contrary, the density of LBE changes as the evolution time increases. After long-term evolution, the density of the LBE samples generally decreased, leading to volume expansion after LBE solidification. The reason for the difference in density between the two is that pure lead is a single metal and does not form a eutectic phase with other metals. During cooling and subsequent static evolution processes, no phase transition occurs, so its macroscopic density does not show significant fluctuations. The significant change in density of LBE is fundamentally due to a phase transition occurring within it. As shown in Figure 1, the microstructure of LBE is composed of γ-phase (bismuth rich phase) and β-phase (Pb_7_Bi_3_); during the evolution process after solidification, the β-phase gradually transforms into the γ-phase. Due to the lower density of the γ-phase compared to the β-phase, the formation of more γ-phase requires a larger space occupation, resulting in a significant change in the overall density of LBE accompanied by volume expansion effects. When the reactor experiences an accident condition or shutdown, the system temperature will suddenly drop, and the excellent density stability exhibited by pure lead is of great significance. Thanks to its absence of phase transition during cooling solidification and subsequent evolution, and its density remaining relatively constant, it can effectively avoid volume changes caused by drastic fluctuations in material density, thereby reducing the risk of stress mutations on the reactor vessel and related components, and helping to improve the operational safety and structural stability of the reactor under accident conditions.

### 3.2. Microstructure Characterization

**Figure 4:**
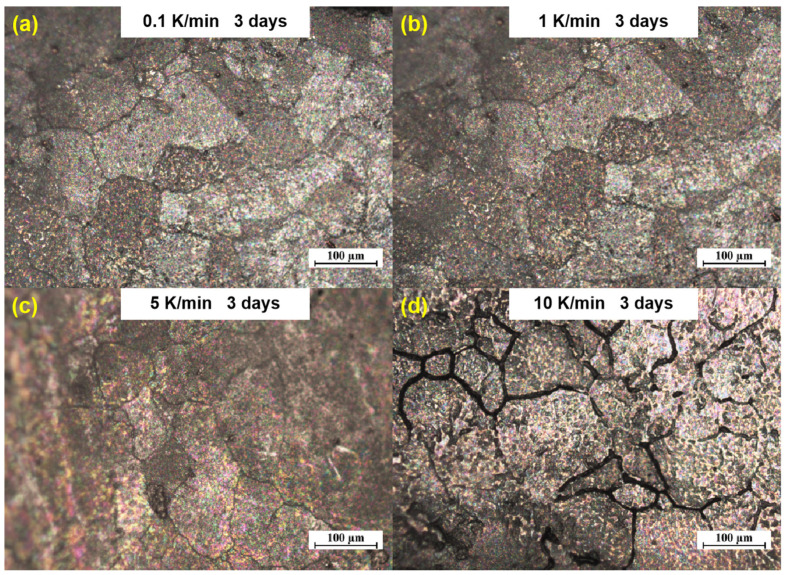
Optical microscope images of the microstructure evolution of pure lead samples after solidification for 3 days at different cooling rates. (**a**) 0.1 K/min pure lead samples have been completed for 3 d, (**b**) 1 K/min pure lead samples have been completed for 3 d, (**c**) 5 K/min pure lead samples have been completed for 3 d, (**d**) 10 K/min pure lead samples have been completed for 3 d.

**Figure 5:**
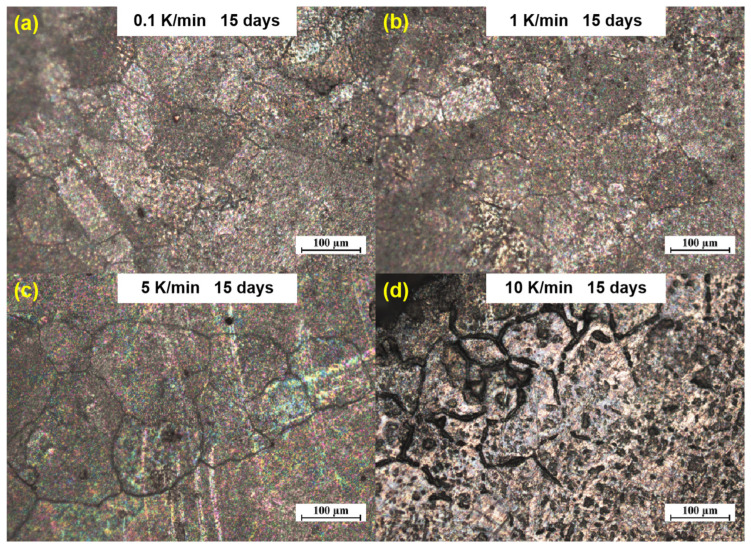
Optical microscope images of the microstructure evolution of pure lead samples after solidification for 15 days at different cooling rates. (**a**) 0.1 K/min pure lead samples have been completed for 15 d, (**b**) 1 K/min pure lead samples have been completed for 15 d, (**c**) 5 K/min pure lead samples have been completed for 15 d, (**d**) 10 K/min pure lead samples have been completed for 15 d.

**Figure 6:**
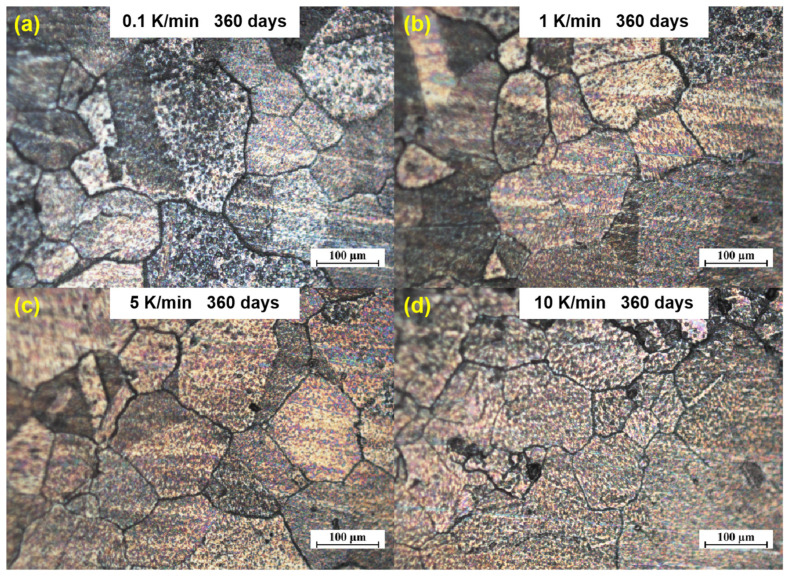
Optical microscope images of the microstructure evolution of pure lead samples after solidification for 360 days at different cooling rates. (**a**) 0.1 K/min pure lead samples have been completed for 360 d, (**b**) 1 K/min pure lead samples have been completed for 360 d, (**c**) 5 K/min pure lead samples have been completed for 360 d, (**d**) 10 K/min pure lead samples have been completed for 360 d.

This study systematically investigated the microstructural evolution of pure lead samples under different cooling rates during long-term static processes (3 d, 15 d, 360 d). Figure 4, Figure 5 and Figure 6 shows the optical microscopy characterization results of pure lead samples at different static stages. It can be seen from the figure that each sample has similar microstructural features. They demonstrate the surface characteristics in polished state and the microstructure of grain boundaries and grains after chemical corrosion. The image presents a typical polycrystalline structure, with grain boundaries clearly visible due to etching, and an overall polygonal network distribution.

Comparing the microstructures under two cooling rates of 0.1 K/min and 10 K/min in Figure 4, it can be clearly seen that the cooling rate has a significant effect on grain size: the faster the cooling rate, the smaller the obtained grains. The main reason is that the faster the cooling rate, the greater the degree of undercooling, making it easier for pure lead to form crystal nuclei during solidification, resulting in smaller grain sizes. This study further analyzed the microstructure evolution of pure lead samples prepared at different cooling rates during long-term static processes (15 d, 360 d), as shown in Figure 5 and Figure 6. Comparing Figure 4 with Figure 5, it can be seen that within only 12 days of static evolution, pure lead grains exhibit a faster growth rate; comparing Figure 4 and Figure 6, it can be observed that after a long period of evolution, the microstructure of pure lead gradually stabilizes, and the difference in grain size under different cooling conditions significantly decreases. The results showed that pure lead samples prepared at different cooling rates exhibited significant grain coarsening during the static process, and the microstructure became more uniform. From the perspective of physical metallurgical mechanisms, pure lead spontaneously undergoes recovery and recrystallization processes at room temperature (27 °C). Driven by the thermodynamic reduction of the free energy at the grain boundary interface, atoms undergo diffusion, grain boundaries undergo migration, and large grains gradually grow by engulfing small grains. After 360 days of static storage, the grain size of samples with different cooling rates tended to be consistent, indicating that the recovery and recrystallization processes were basically completed, and the microstructure of pure lead samples reached a relatively stable state. During the microscopic characterization process, all optical micrographs observed surface features after oxidation and etching. Due to the easy oxidation of pure lead, after long-term polishing and etching treatment, an oxide film is quickly formed on the surface of the sample in the air, and the etching process also introduces local slight surface defects. However, the above-mentioned oxidation and surface defects are only distributed on the surface of the sample, and have no substantial effect on the observation of grain size and microscopic characteristics of grain boundaries. Therefore, they do not interfere with the core characterization and analysis of grain size and microstructure evolution in this study.

**Figure 7:**
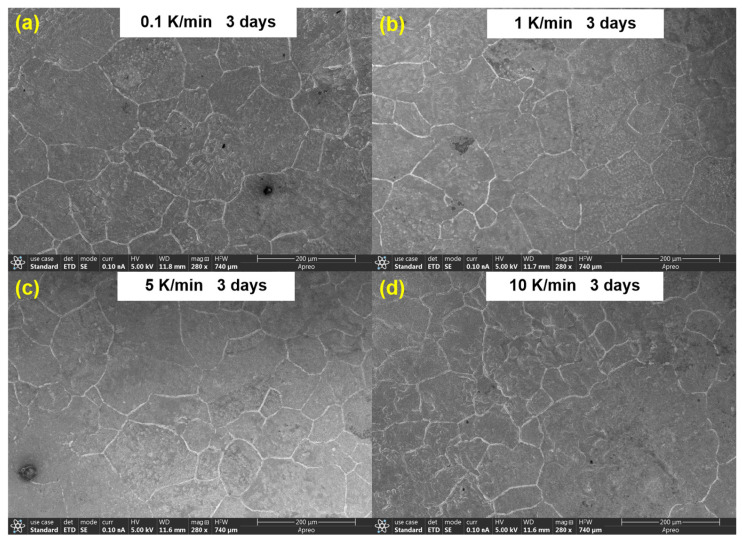
SEM images of the microstructure evolution of pure lead samples after solidification for 3 days at different cooling rates. (**a**) 0.1 K/min pure lead samples have been completed for 3 d, (**b**) 1 K/min pure lead samples have been completed for 3 d, (**c**) 5 K/min pure lead samples have been completed for 3 d, (**d**) 10 K/min pure lead samples have been completed for 3 d.

**Figure 8:**
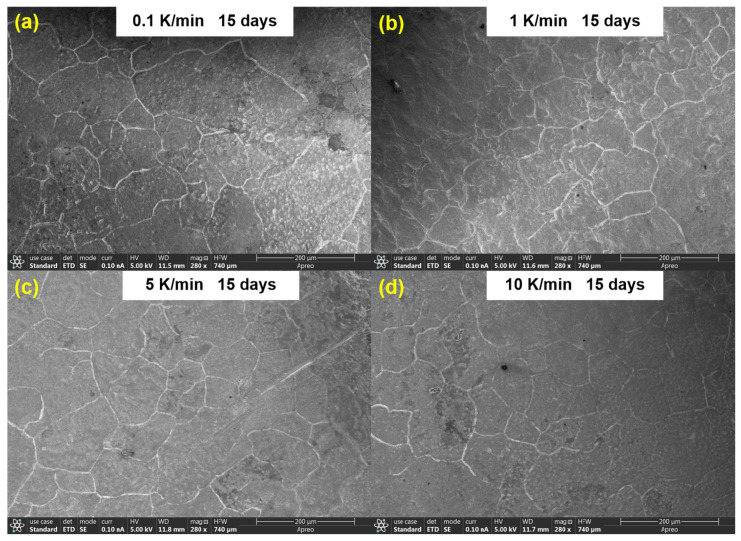
SEM images of the microstructure evolution of pure lead samples after solidification for 15 days at different cooling rates. (**a**) 0.1 K/min pure lead samples have been completed for 15 d, (**b**) 1 K/min pure lead samples have been completed for 15 d, (**c**) 5 K/min pure lead samples have been completed for 15 d, (**d**) 10 K/min pure lead samples have been completed for 15 d.

**Figure 9:**
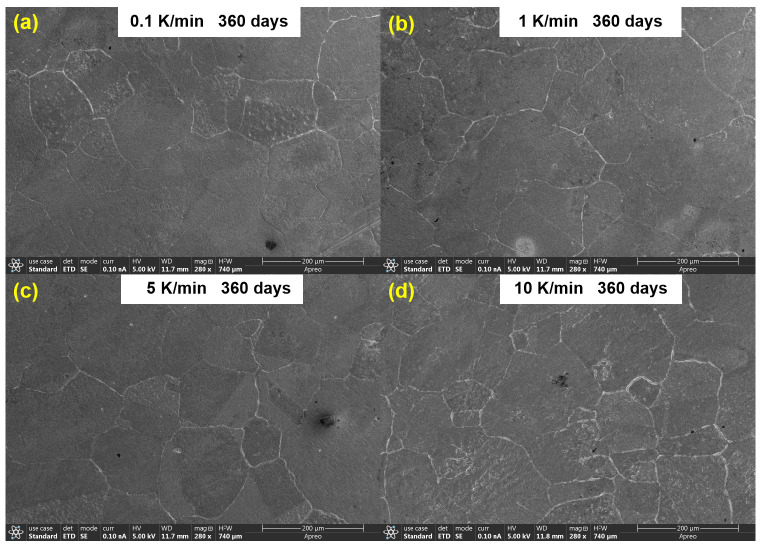
SEM images of the microstructure evolution of pure lead samples after solidification for 360 days at different cooling rates. (**a**) 0.1 K/min pure lead samples have been completed for 3 d, (**b**) 1 K/min pure lead samples have been completed for 360 d, (**c**) 5 K/min pure lead samples have been completed for 360 d, (**d**) 10 K/min pure lead samples have been completed for 360 d.

To more accurately reveal the microstructural evolution details of pure lead samples during long-term standing, this study further characterized the samples using scanning electron microscopy (SEM), as shown in Figure 7, Figure 8 and Figure 9. The SEM observation results are highly consistent with the optical microscope characterization results, verifying the reliability of the microstructure evolution law. Based on SEM images, this study further quantitatively characterized the grain size of pure lead: pure lead grains exhibit a nearly polygonal characteristic as a whole. The IPS algorithm was used to statistically calculate the grain size under different cooling rates and standing times, and the results are shown in Table 1.

### 3.3. Mechanical Property

**Figure 10:**
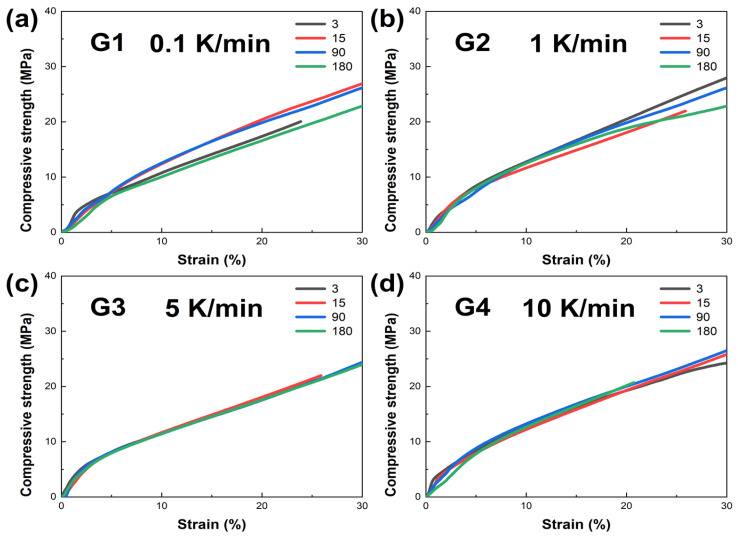
Compression mechanical curves of the pure lead samples. (**a**) 0.1 K/min pure lead samples have been completed for 3~180 d, (**b**) 1 K/min pure lead samples have been completed for 3~180 d, (**c**) 5 K/min pure lead samples have been completed for 3~180 d, (**d**) 10 K/min pure lead samples have been completed for 3~180 d.

This study conducted compressive mechanical property tests on pure lead samples obtained at different cooling rates, and obtained the compression mechanical curves of pure lead samples during long-term evolution (3~180 days). The results are shown in Figure 10. The curves of different colors in the figure represent the test results at different times (3, 15, 90, and 180 days).

In Figure 10a, it can be observed that at a cooling rate of 0.1 K/min, the elastic deformation stage of the pure lead sample is very short, quickly reaching the yield point and entering the plastic deformation stage. Further observation of the plastic deformation stage indicates that when the strain reaches 20%, the compression mechanical curve of the sample continues to increase and no fracture occurs. This indicates that pure lead samples have relatively low strength and are prone to deformation in compressive mechanical tests, but have good toughness. The compression mechanical curves in Figure 10b–d show a similar trend.

As the evolution time increases, the compression mechanical curve shows a gradual downward trend. This indicates that, under the same cooling rate, the compressive strength of the pure lead sample gradually decreases as the evolution time is extended.

**Figure 11:**
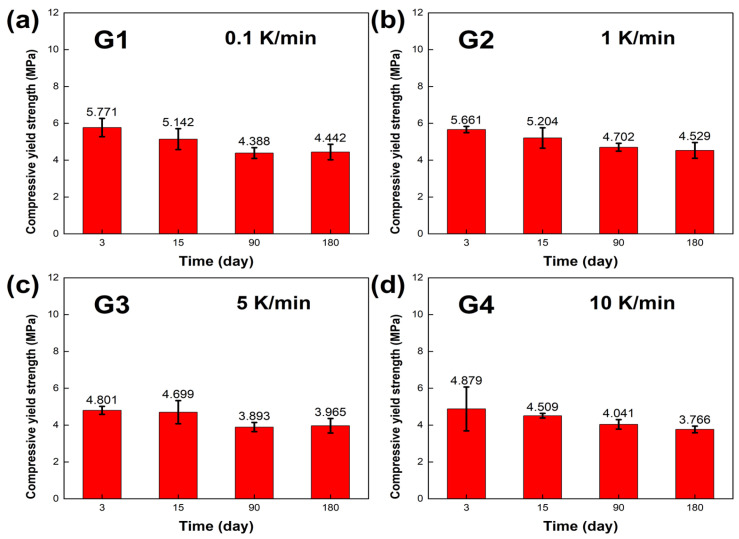
Compressive yield strength (σ) of the pure lead samples. (**a**) 0.1 K/min pure lead samples have been completed for 3~180 d, (**b**) 1 K/min pure lead samples have been completed for 3~180 d, (**c**) 5 K/min pure lead samples have been completed for 3~180 d, (**d**) 10 K/min pure lead samples have been completed for 3~180 d.

The compressive yield strength (σ) of pure lead has been obtained, as shown in Figure 11. Under a compression rate of 10^−3^ s^−1^ and at room temperature, the compressive yield strength (σ) of the pure lead samples ranged between 3 and 6 MPa.

Figure 11a shows the compressive yield strength (σ) at a cooling rate of 0.1 K/min, and the compressive yield strength (σ) of the sample shows a gradually decreasing trend with increasing evolution time (3~180 days). As the cooling rate increases, further observation of Figure 11b–d reveals that this trend also occurs in other cooling rates. By comparison, it can be observed that in the initial stage after solidification, samples using rapid cooling processes (5 K/min and 10 K/min) have lower yield strength than samples using slow cooling processes (0.1 K/min and 1 K/min).

According to the Hall–Petch type inference, the yield strength of materials is closely related to grain size. The larger the grain size, the weaker the hindrance effect of grain boundaries on dislocation movement. Dislocations are more likely to slip inside the grain and pass through the grain boundaries, resulting in a decrease in macroscopic yield strength. Pure lead atoms have strong diffusion ability. After preparation, the sample is stored in a vacuum box at room temperature (about 27 °C), which happens to be in the temperature range where lead is prone to recovery and recrystallization. Therefore, recovery and recrystallization will continue to occur, accompanied by continuous grain coarsening. As the grain size gradually increases, the proportion of grain boundaries decreases, and the constraint effect on dislocation motion is correspondingly weakened, ultimately resulting in a significant decrease in the yield strength of the material with the coarsening of the microstructure. This correlation is consistent with the microscopic characterization results mentioned earlier: after long-term evolution of pure lead solidification, its internal grain size will increase, leading to a decrease in its yield strength.

The main reason for the difference in the evolution of yield strength between samples cooled quickly and slowly is that the samples under both cooling regimes undergo recovery, recrystallization after solidification, and show a trend of decreasing yield strength; However, compared to slow cooling, rapid cooling results in smaller initial grain size, higher internal defect density, and residual stress, which enhances the thermodynamic driving force for subsequent recovery and recrystallization, and leads to a more significant degree of grain coarsening. Therefore, the yield strength of rapidly cooled samples decreases more significantly during the subsequent tissue evolution process, and the magnitude of strength change is greater than that of slowly cooled samples. However, the law of reduced yield strength due to grain coarsening in pure lead is currently only at the qualitative description level, and a direct quantitative correspondence between grain size and yield strength has not yet been established.

Additionally, to verify the reliability and accuracy of the obtained compressive yield strength (σ) results for the pure lead samples in this study, repetitive compressive mechanical tests were conducted. Through multiple repetitions of the experiments, the obtained error results were within an acceptable range. The results indicate that the experimental methods and data processing procedures used in this study are stable and reproducible, and the obtained compressive yield strength (σ) results for pure lead exhibit good accuracy and credibility.

As promising coolant materials for Generation IV nuclear reactors, such as Lead-cooled Fast Reactors (LFRs), pure lead and LBE (lead–bismuth eutectic) are a focal point of current nuclear energy research. These systems are designed for long-term operation in harsh service environments characterized by high temperatures, intense radiation, and severe thermal cycling. Consequently, the mechanical properties of these materials—particularly their yield strength, which is the cornerstone of structural integrity—are directly critical to the safe operation of the entire reactor. When a reactor accident or shutdown occurs, its internal temperature will sharply drop, and LBE and pure lead will gradually solidify, which is likely to affect the microstructure and mechanical properties of the two materials after solidification.

**Figure 12:**
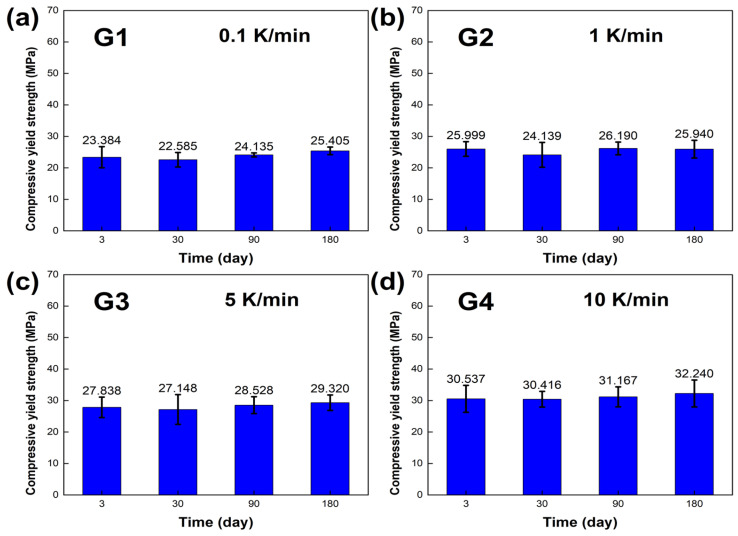
Compressive yield strength (σ) of the LBE samples obtained. (**a**) 0.1 K/min LBE samples have been completed for 3~180 d, (**b**) 1 K/min LBE samples have been completed for 3~180 d, (**c**) 5 K/min LBE samples have been completed for 3~180 d, (**d**) 10 K/min LBE samples have been completed for 3~180 d [31].

Prior to this, we conducted studies on the long-term evolution characteristics of LBE samples with different cooling rates, and obtained the results of the compressive yield strength of LBE after solidification for 3 to 180 days, as shown in Figure 12 [31]. A comparative study was conducted on the yield strength of pure lead and LBE samples during the long-term evolution period after solidification, under the premise of completely consistent sample preparation, cooling method, processing technology, and storage environment. In Figure 12, it can be seen that as the evolution time increased, there was no significant difference in the compressive yield strength (σ) of the LBE samples with the same cooling rate and only a certain degree of fluctuation, indicating that evolution time has a minor effect on the compressive yield strength (σ) of the LBE. However, with the increase in cooling rate, the compressive yield strength (σ) shows an upward trend [31]. The experimental results indicate that during the long-term evolution process after solidification, evolution time had no significant effect on the yield strength of LBE. On the contrary, the yield strength of pure lead shows a gradually decreasing trend. Moreover, the cooling rate has a significant impact on the yield strength of both materials. These results profoundly demonstrate the crucial influence of cooling conditions and evolution time on the final mechanical properties of LBE and pure lead. Therefore, in engineering applications, it is imperative to strictly control their solidification processes.

## 4. Conclusions

In this study, pure lead samples were prepared at different cooling rates. The long-term evolution of the microstructure and mechanical properties of the solidified pure lead was systematically investigated, and compared and analyzed with LBE. The main conclusions are as follows:

(1) The density of pure lead samples obtained under different cooling rates showed no significant change during the long-term evolution. In contrast, after long-term evolution, the LBE sample undergoes a phase transition internally, leading to a decrease in density and accompanied by volume expansion behavior.

(2) After solidification, pure lead can spontaneously undergo recovery and recrystallization processes at room temperature (27 °C). After long-term structural evolution, its grains will further coarsen significantly, and its microstructure will tend to be more uniform; in contrast, LBE undergoes a phase transition during the long-term aging process after solidification, from the β-phase to the γ-phase.

(3) During the long-term evolution after solidification, the evolution time has no significant effect on the yield strength of LBE. On the contrary, pure lead will coarsen its grains after long-term evolution, leading to a gradual decrease in yield strength. The cooling rate has a significant impact on the yield strength of both materials. Therefore, in engineering applications, it is imperative to strictly control their solidification processes.

(4) The long-term evolution laws of the microstructure, density, and mechanical properties of pure lead after solidification have been clarified, filling the gap in the long-term basic data of pure lead in lead based fast reactor coolant, and revealing the essential differences in the long-term evolution mechanisms of pure lead and LBE after solidification. The excellent density stability of pure lead can avoid structural risks caused by solidification volume expansion under shutdown/accident conditions, providing a key basis for the safety design of reactor vessels and circuits.

(5) This study only examined the long-term evolution under static conditions at room temperature, and the characterization methods and experimental conditions covered were relatively limited. In the future, characterization methods can be further enriched, and key experimental conditions such as cooling rate, storage temperature, and time period can be expanded to reveal more in-depth the microstructure and performance evolution laws of pure lead.

## Figures and Tables

**Table 1:** Average grain size of samples with different cooling rates.

Temperature (K/min)	Average Grain Size over 3 Days (µm)	Average Grain Size over 15 Days (µm)	Average Grain Size over 360 Days (µm)
0.1	68.958 ± 39.121	71.408 ± 37.422	112.497 ± 24.415
1	65.957 ± 21.565	70.400 ± 28.948	108.997 ± 26.947
5	62.782 ± 30.329	74.774 ± 20.279	115.172 ± 33.638
10	60.149 ± 22.167	79.201 ± 16.954	114.12 ± 37.625

## Data Availability

The data presented in this study are available on request from the corresponding author due to the privacy protection requirements of the funding party (Science and Technology on Reactor System Design Technology Laboratory). The data contains technical information related to reactor system design that is not publicly available.
